# Comparison of cardiac volumetry using real-time MRI during free-breathing with standard cine MRI during breath-hold in children

**DOI:** 10.1007/s00247-022-05327-5

**Published:** 2022-03-30

**Authors:** Lena Maria Röwer, Karl Ludger Radke, Janina Benner, Halima Malik, Tobias Uelwer, Dirk Voit, Jens Frahm, Hans-Joerg Wittsack, Stefan Harmeling, Frank Pillekamp, Dirk Klee

**Affiliations:** 1 Department of General Pediatrics, Neonatology and Pediatric Cardiology, University Children’s Hospital, Moorenstr. 5, 40225 Dusseldorf, Germany; 2 Department of Diagnostic and Interventional Radiology, Medical Faculty, Heinrich Heine University, Dusseldorf, Germany; 3 Department of Computer Science, Heinrich Heine University, Dusseldorf, Germany; 4 Biomedizinische NMR, Max-Planck-Institut für biophysikalische Chemie, Göttingen, Germany; 5 DZHK (German Centre for Cardiovascular Research), Göttingen, Germany

**Keywords:** Computer-assisted, Children, Heart, Image processing, Magnetic resonance imaging, Respiration, Volumetry

## Abstract

**Background:**

Cardiac real-time magnetic resonance imaging (RT-MRI) provides high-quality images even during free-breathing. Difficulties in post-processing impede its use in clinical routine.

**Objective:**

To demonstrate the feasibility of quantitative analysis of cardiac free-breathing RT-MRI and to compare image quality and volumetry during free-breathing RT-MRI in pediatric patients to standard breath-hold cine MRI.

**Materials and methods:**

Pediatric patients (*n* = 22) received cardiac RT-MRI volumetry during free breathing (1.5 T; short axis; 30 frames per s) in addition to standard breath-hold cine imaging in end-expiration. Real-time images were binned retrospectively based on electrocardiography and respiratory bellows. Image quality and volumetry were compared using the European Cardiovascular Magnetic Resonance registry score, structure visibility rating, linear regression and Bland–Altman analyses.

**Results:**

Additional time for binning of real-time images was 2 min. For both techniques, image quality was rated good to excellent. RT-MRI was significantly more robust against artifacts (*P* < 0.01). Linear regression revealed good correlations for the ventricular volumes. Bland–Altman plots showed a good limit of agreement (LoA) for end-diastolic volume (left ventricle [LV]: LoA -0.1 ± 2.7 ml/m^2^, right ventricle [RV]: LoA -1.9 ± 3.4 ml/m^2^), end-systolic volume (LV: LoA 0.4 ± 1.9 ml/m^2^, RV: LoA 0.6 ± 2.0 ml/m^2^), stroke volume (LV: LoA -0.5 ± 2.3 ml/m^2^, RV: LoA -2.6 ± 3.3 ml/m^2^) and ejection fraction (LV: LoA -0.5 ± 1.6%, RV: LoA -2.1 ± 2.8%).

**Conclusion:**

Compared to standard cine MRI with breath hold, RT-MRI during free breathing with retrospective respiratory binning offers good image quality, reduced image artifacts enabling fast quantitative evaluations of ventricular volumes in clinical practice under physiological conditions.

**Supplementary Information:**

The online version contains supplementary material available at https://doi.org/10.1007/s00247-022-05327-5.

## Introduction

The importance of cardiac magnetic resonance imaging (MRI) in pediatric cardiology as a noninvasive diagnostic method has increased substantially over the last few years. Cardiac MRI permits the flexible choice of imaging planes [1] that is relevant for the anatomical presentation of the heart and the quantification of functional parameters, such as ventricular volumetry [2–4]. Nevertheless, balanced standard steady-state free precession (bSSFP) cine MRI is associated with some disadvantages, such as the occasional need for data interpolation to display a heartbeat due to a low temporal resolution and the need for repetitive breath-hold maneuvers to achieve high image quality. This reduces the patient’s comfort [5] and complicates the use in young pediatric patients.

Real-time (RT)-MRI offers unique advantages for the representation of the heart: It provides accelerated imaging by radial data encoding in combination with nonlinear inverse reconstruction [6–8]. Thus, RT-MRI enables the acquisition of up to 50 single images per second [6]. This advanced imaging technique significantly increases temporal resolution and robustness against motion artifacts, which translates into improved image quality. Since cardiac RT-MRI allows for data acquisition during free breathing, it greatly enhances the convenience of cardiac MRI for all patients and simplifies the investigation of young pediatric patients. Furthermore, the examination takes place under physiological conditions, which improves the informative value of cardiac function regarding heart–lung interactions [9, 10].

Although cardiac RT-MRI offers tremendous advantages in the qualitative representation of the heart, the lack of quantitative evaluation strategies precludes its current use in clinical practice. The aim of this study was (1) to enable the quantitative analysis of cardiac RT-MRI volumetry in pediatric patients for daily clinical use by combining RT-MRI during free breathing with retrospective binning of images by electrocardiography (ECG) and respiratory bellows and (2) to evaluate ventricular function parameters and image quality by comparing the RT-MRI results to those obtained by standard cine MRI. To this end, we hypothesized that the combination of cardiac real-time MRI during free breathing in pediatric patients with retrospective respiratory binning offers good image quality, reduced image artifacts and quantitative evaluations of ventricular volumes.

## Materials and methods

### Experimental design

The study was designed as a descriptive, observational in vivo imaging study of pediatric patients recruited from the Department of Pediatric Cardiology at the University Hospital Duesseldorf and was approved by the local ethics committee (Ethics Committee of the Medical Faculty, University Hospital Duesseldorf, Germany, study number 6176R). Written informed consent was obtained from the children’s parents or their legal guardians before the study.

Body surface was calculated using the DuBois formula with 1.6 m^2^ ± 0.4 m^2^ and used to calculate the left ventricle (LV) and right ventricle (RV) indexed volume results.

MRI measurements were performed on a clinical 1.5-T MRI scanner (MAGNETOM Avanto Fit; Siemens Healthineers, Erlangen, Germany; software version syngo MR E11). For the measurements, an MR table with an installed 32-channel spine matrix coil (direct connect spine 32, Siemens Healthineers, Erlangen, Germany) was used, and an 18-channel body coil (Body 18, Siemens Healthineers, Erlangen, Germany) was placed around the patients.

The clinical cardiac MRI examination included standard cine MRI volumetry (i.e., multiple cross-sections) with bSSFP contrast, retrospective gating and segmented k-space-filling (Table 1). Repetitive breath-holding maneuvers with durations of 7–10 s were required for the acquisition of each slice of the short axis stack, covering the whole heart. The slice orientation of the short axis stack was perpendicular to the interventricular septum. The number of slices (1 × 10 slices [excluded patient], 2 × 13 slices; 7 × 14 slices; 10 × 16 slices, 2 × 18 slices) and the field of view (FOV) were determined individually depending on the patient’s heart size (Table 1). The slices were acquired with a thickness of 6 mm (in 4 patients) or 8 mm (in 18 patients) and without a gap between image slices. Within the cardiac cycle, images were acquired at 25 different time points.

**Table 1:** Detailed sequence parameters for standard cine MRI and RT-MRI

Sequence parameters	Cine MRI	RT-MRI
Sequence type	2D b-SSFP	2D b-SSFP
TR/TE (ms)	58.3/1.1	3.7/1.9
FOV (mm)	316–500	316–500
Image matrix (pixels)	192	200
Pixel size (mm/pixel)	1.9 × 1.9x8.0	1.6 × 1.6x8.0
Slices (n)	10–18	10–18
Slice thickness (mm)	6–8	6–8
Interslice gap (mm)	0	0
Phases	25	900
Orientation	Short axis	Short axis
Flip angle (°)	80	60
Bandwidth (Hz)	930	760
ECG synchronization	Retrospective	-

*b-SSFP* balanced steady-state free precession *ECG* electrocardiography, *FOV* field of view, *RT-MRI* real-time magnetic resonance imaging, *TE* echo time, *TR* repetition time

The clinical cardiac MRI examination was followed by cardiac RT-MRI volumetry with bSSFP contrast (33 ms per image) (Table 1). Slice orientation and imaging parameters were identical to the standard sequence. RT-MRI data were acquired continuously during free breathing at a rate of 30 images per s over a duration of 30 s for each slice. ECG and the respiratory bellows signal for detecting the respiratory movement were recorded via Siemens Signal logging VE11a (Siemens Healthineers, Erlangen, Germany).

### Real-time data processing

**Fig. 1:**
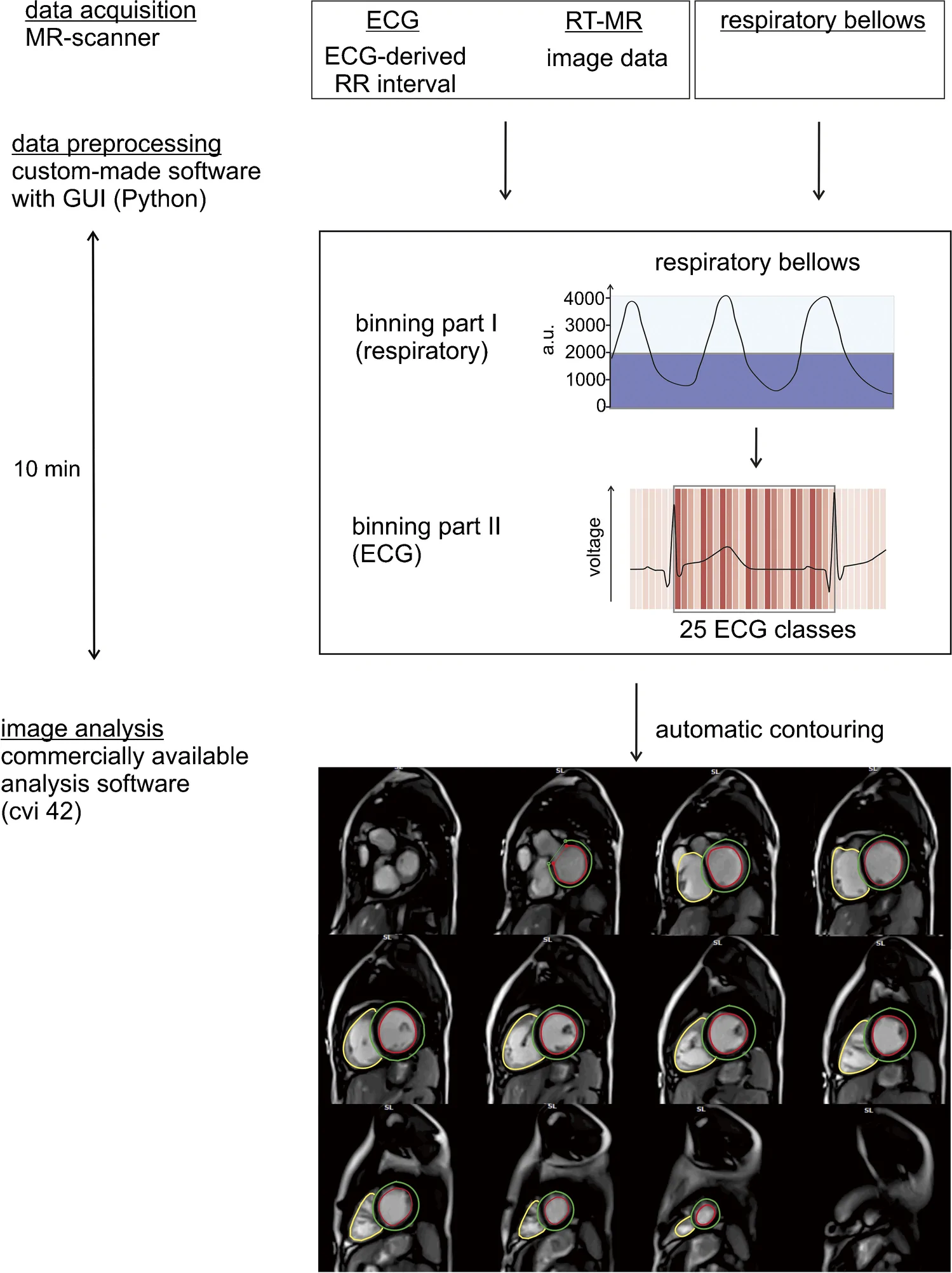
RT-MRI processing. RT images in short axis orientation included the ECG derived time after the R-peak in their DICOM tags. A custom-made software program assigned the respiratory bellows values to the DICOM tags. Images with respiratory bellows values corresponding to a low lung volume (respiratory bellows values < 2000 a.u.) were binned into 25 ECG classes. RT images (from RT balanced SSFP sequence, planes 5-16) were imported into a commercially available analysis software (cvi42) for contouring. *a.u*. arbitrary units, *cvi* cardiovascular imaging, *DICOM* Digital Imaging and Communications in Medicine, *ECG* electrocardiography, *RT* real-time, *RT-MRI* real-time magnetic resonance imaging*, SSFP* steady-state free precession

The RT-MR images were binned based on respiration and ECG-derived RR intervals using an in-house developed software with graphical user interface (GUI) based on programming in Python (v3.8.4. Python Software Foundation, Wilmington, DE) by adapting published open-source packages (e.g., Numpy [11] and pydicom [12]. For details on programming, see https://doi.org/10.5281/zenodo.6352262. The individual ECG-derived time after the R-wave was provided by the intrinsic MR scanner software (syngo MR E11; Siemens Healthineers, Erlangen, Germany) and included in the DICOM tags from the RT-MR images (Fig. 1). Information on the respiratory phase obtained by respiratory bellows (Fig. 1) underwent typical preprocessing (denoising using a Butterworth-filter, temporal synchronization) and was assigned to the RT images. Thereafter, RT images were binned retrospectively to 25 ECG phases corresponding to a low lung volume (respiratory bellows < 2,000 a.u.) using ECG and respiratory bellows (Fig. 1). The duration of the 25 phases of a heart cycle was individually chosen for each patient considering the mean RR interval (Table 2). In the case of more than one provided image per ECG class, the image closest to the median of the respiratory class (respiratory bellows = 1,000 a.u.) was chosen. After automatic binning, the RT-MR images were imported into the commercial evaluation software cvi42 (Release 5.10.1.[1241]; Circle Cardiovascular Imaging Inc., Calgary, Canada) (Fig. 1).

**Table 2:** Patient information

Patient information							Indication for cardiac MRI	
Patient	Age (years)	Gender	Body weight (kg)	Body height (cm)	BSA (DuBois) (m^2^)	RR interval (ms)	Cardiac disease	Main question
1	13	Female	52	160	1.5	700	D-transposition of the great arteries	Ventricular function, neo-aortic regurgitation
2	15	Male	64	178	1.8	750	Atrioventricular septal defect	Atrial and ventricular volumetry
3	8	Female	23	124	0.9	640	Tetralogy of Fallot	RV function and volumetry
4	7	Female	22	122	0.9	500	Truncus arteriosus	RV function and volumetry
5	14	Male	70	189	2.0	990	Hypertrophic cardiomyopathy	Ventricular function, myocardial mass
6	10	Female	65	153	1.6	850	Hypertrophic obstructive cardiomyopathy	Ventricular function, myocardial mass
7	17	Male	42	167	1.4	525	Myocarditis	Ventricular function, myocardial late enhancement
8	17	Male	94	193	2.3	950	Aortic stenosis and regurgitation	LV function and volumetry
9	11	Female	30	153	1.2	700	D-transposition of the great arteries	Ventricular function, pulmonary flow
10	15	Female	55	154	1.5	875	Double outlet RV, pulmonary stenosis	RV function and volumetry
11	11	Male	49	172	1.6	740	Aortic regurgitation	LV function and volumetry
12	14	Male	78	187	2.0	700	Total anomalous pulmonary venous return	Ventricular function, pulmonary venous stenosis
13	17	Male	55	160	1.6	900	Congenital aortic stenosis	LV function and volumetry
14	17	Male	92	180	2.1	840	Myocarditis	Ventricular function, myocardial late enhancement
15	10	Male	48	146	1.4	750	Atrial septal defect, Coronary artery anomaly	Ventricular function, anatomical representation of coronary arteries
16	10	Male	33	133	1.1	530	Chemotherapy-induced myocardial dysfunction	LV function and volumetry
17	14	Female	64	165	1.7	750	Atrioventricular septal defect	LV function and volumetry
18	16	Male	58	181	1.8	800	Patent foramen ovale	RV function and volumetry, pulmonary and systemic flow
19	5	Female	15	101	0.6	650	Double outlet RV	RV function and volumetry, conduit assessment
20	15	Male	85	183	2.1	800	Non-compaction cardiomyopathy	LV function and volumetry
mean ± SD	12.8 ± 3.5	Male = 12 Female = 8	54.7 ± 22.3	160.1 ± 24.3	1.6 ± 0.4	747.0 ± 132.3	-	-

Detailed presentation of patient characteristics and indications for cardiac MRI

*BSA* body surface area, *LV* left ventricle/left ventricular, *RR interval* interval between two consecutive R waves on the electrocardiogram, *RV* right ventricle/right ventricular, *SD* standard deviation

### Data analysis

The cine short axis stack in end-expiratory breath holding and the RT short axis stack acquired during free breathing and binned corresponding to a low lung volume were analyzed exactly in the same way, using cvi42. Image quality, LV and RV volumes were evaluated. Image contrast of the cine and RT images differed when standard windowing was used. Visual image contrast was adapted by using auto windowing type 2 for RT-MRI, provided by the evaluation software, to provide a fair comparison. The visual image contrast of RT-MRI was adjusted using a thresholding algorithm based on the histogram of pixel signal intensity values. A threshold count was calculated as (threshold count = total pixels in image/2,500). The threshold signal intensity was then determined as the most considerable signal intensity value in the histogram with at least threshold count pixels. Based on the threshold signal intensity, the window width controlling for visual image contrast was calculated as (window width = threshold signal intensity—minimal signal intensity) and the window center controlling for the image brightness was calculated as (window center = [window width/2] + minimal signal intensity).

### Image quality assessment

The image quality of cardiac RT-MRI and cine MRI was compared by D.K., a pediatric radiologist with 16 years of experience in cardiovascular MRI and L.M.R. (radiologic assistant in the first year) using two established cardiac MRI scores. The European cardiac MRI score was used to rate artifacts (wraparound, respiratory ghosting, cardiac ghosting, blurring/miss-triggering, metallic artifact, shimming) on MR images [13, 14]. The short axis stacks from cardiac RT-MRI and cine MRI were rated from 0 to 3 with 0 = no artifacts, 1 = artifacts on one slice, 2 = artifacts on two slices, and 3 = artifacts on three or more slices. In addition, the structure visibility on the end-diastolic and end-systolic phases from RT and cine MRI were evaluated based on 4-point scales [13, 15]. The visibility of LV and RV endocardial borders, LV epicardial borders, papillary muscles, blood pool contrast, myocardium and cardiac motion was rated on a scale of 1 = no visibility, 2 = poor visibility, 3 = good visibility and 4 = excellent visibility. Inter-rater reliability was determined using the evaluations of D.K. and L.M.R. To obtain intra-rater reliability, L.M.R. repeated the image quality analysis at least 4 weeks after the initial assessment. The mean values of the three ratings were calculated and used for further analysis.

### Volumetry analysis

The short 3-D module of cvi42 was used to automatically contour the LV endocardial and epicardial contours and the RV endocardial contours at end-diastole and end-systole. L.M.R. performed manual corrections based on a standardized approach at the Children’s University Hospital, University Hospital Duesseldorf, Germany [9] that was established considering current recommendations on cardiac image analysis [16–19]. To test intra- and inter-rater reliability for the LV and RV volume evaluation, L.M.R. and J.H. (fifth-year medical student) repeated the volumetric analysis at least 4 weeks after the first measurement in 30% randomly chosen patients (*n* = 7).

### Statistical analysis

All statistical analyses were performed in SPSS (SPSS Statistics for Windows, Version 26.0; IBM Corp., Armonk, NY). The Shapiro–Wilk test was used to test for normal distribution. In the case of normal distributed data, e.g., ventricular function parameters, a paired sample *t*-test was calculated. The Wilcoxon rank sum test was used in the case of non-normal distributed and ordinal-scaled data, e.g., image quality analysis. Linear regression analyses were performed and Bland–Altman plots were generated to evaluate the agreement between LV and RV function parameters obtained with RT and cine MRI. The intra-rater and inter-rater reliability for the volumetric analysis was determined with the intraclass correlation coefficient for the ICC and was classified as poor (ICC < 0.5), fair (0.5 < ICC < 0.75), good (0.75 < ICC < 0.9) and excellent (ICC > 0.9) [20]. Spearman rho rank correlation (*P*) was used to determine intra- and inter-rater reliability in the case of ordinal-scaled data, e.g., image quality analysis. The effect size rho was classified as small (0.1–0.3), medium (0.3–0.5) and strong (> 0.5), according to Cohen [21]. A significance level of *P* < 0.05 was considered statistically significant.

## Results

### Patient details

Twenty pediatric patients (12 male, 8 female) from the 22 recruited patients were included in the study (Table 2). Two patients were excluded, one due to MRI termination on the patient's request and the second because of an unusable respiratory bellows signal. Patients had different congenital or acquired heart diseases and had different indications for cardiac MRI (Table 2). The age of the included patients ranged from 5 to 17 years with a mean ± SD age of 12.8 years ± 3.5 years, mean ± SD body weight of 54.7 kg ± 22.3 kg and mean body height of 160.1 cm ± 24.3 cm. Breath holding could not be performed by the three youngest participants ages 5 to 8 years. The mean interval between two R-waves on the electrocardiogram (RR interval) varied widely among the children (747.0 ms ± 132.3 ms) depending on the patient’s age (Table 2).

### Real-time data processing

The use of the software with GUI reduced the manual RT data preparation time for the radiologist to about 2 min. After manual preparation, the RT data processing ran automatically and took about 10 min (on a computer with a Xeon e3 v6 (Intel Corporation, Santa Clara, CA) central processing unit).

### Image quality analysis

#### Reproducibility

Spearman rho rank correlation showed a strong and statistically significant correlation for the structure visualization analysis for both, cine MRI (rho = 0.91, *P* < 0.01) and RT-MRI (rho = 0.95, *P* < 0.01) for inter-rater reliability. Intra-rater reliability was determined for cine MRI with rho = 0.94 (*P* < 0.01) and for RT-MRI with rho = 0.97 (*P* < 0.01). The artifact rating provided strong correlations for cine MRI (rho = 1.0, *P* < 0.01) and RT-MRI (rho = 0.97, *P* < 0.01) for intra-rater reliability and for cine MRI (rho = 0.65, *P* < 0.01) and RT-MRI (rho = 0.98, *P* < 0.01) for inter-rater reliability.

#### Structure visualization rating

**Fig. 2:**
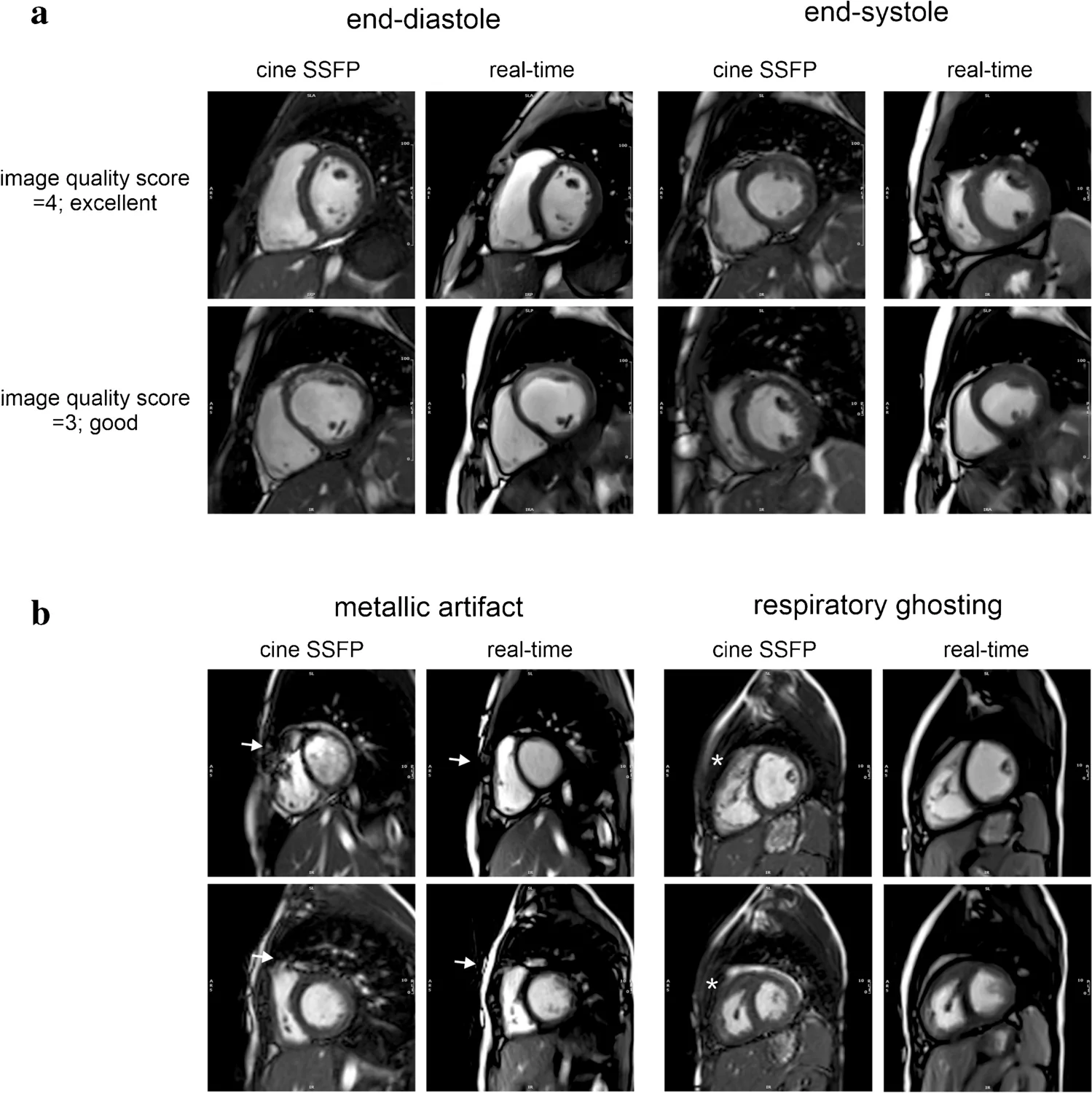
Image quality analysis. **a**, **b** Real time and conventional MR images in short axis orientation. **a** Standard cine images (conventional cine balanced SSFP sequence, planes 6 and 9) and RT images (RT balanced SSFP sequence, planes 6 and 9) of 7-, 14- and 16-year-old patients no. 4, 12, 18, (female, male, male), were rated good = 3 and excellent = 4 on average for structure visualization based on established 4-point scales [13, 15]. **b** The most common artifacts were metallic artifacts (e.g., sternal clips) (*arrows*), which affected standard cine MRI significantly more often and more severely than RT-MRI. Images of 11-year-old patient no. 9 (female). Respiratory artifacts (*stars*) occurred more often with cine MRI whereas RT-MRI was not affected by respiratory ghosting. Images of 5-year-old patient no. 19 (female). *MRI* magnetic resonance imaging, *RT* real-time, *RT-MRI* real-time magnetic resonance imaging, *SSFP* steady-state free precession

The structure visualization assessment of the end-systolic and end-diastolic images using cine MRI and RT-MRI were rated good to excellent on average. Images with poor quality and images in which the structures were not visible did not occur (Fig. 2).

**Fig. 3:**
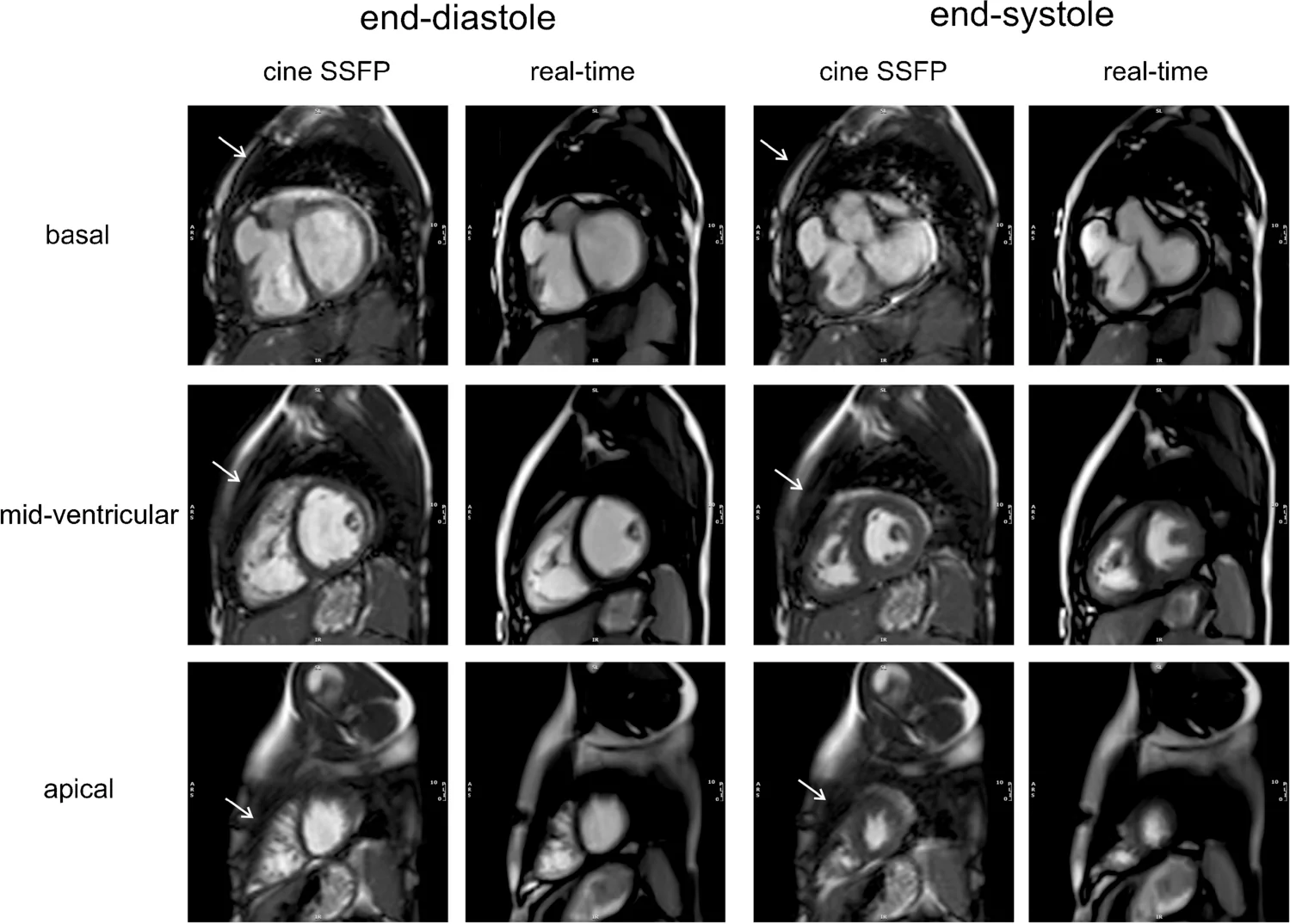
Representative images of the youngest patient, a 5-year-old girl (patient 19) falling asleep during RT-MRI. Images in short axis orientation from basal (plane 8), mid-ventricular (plane 11) and apical (plane 15) levels are presented at end-diastole and end-systole for RT-MRI (RT balanced SSFP sequence) and corresponding cine MRI (conventional cine balanced SSFP sequence). RT-MRI showed excellent image quality. In contrast, cine MRI suffered from respiratory ghosting. Arrows mark respiratory artifacts. *MRI* magnetic resonance imaging, *RT-MRI* real-time magnetic resonance imaging*, SSFP* steady-state free precession

Cine images were slightly superior to RT images in most structural visualization categories, especially in patients with good breath hold (Table 3 and supporting video in Online Supplementary Material 1, Part 1). Minor differences were seen in LV/RV endocardial borders (cine: 3.7 ± 0.4; RT: 3.5 ± 0.5), epicardial borders (cine: 3.7 + /0.4; RT: 3.5 ± 0.5) and myocardium (cine: 3.8 ± 0.3; RT: 3.6 ± 0.5). Cardiac motion showed no difference between both techniques (cine: 3.9 ± 0.2; RT: 3.9 ± 0.2). Whereas especially the visualization of the papillary muscles (cine: 3.9 0.2; RT: 3.5 ± 0.5) and the blood pool contrast (cine: 3.9 ± 0.3; RT: 3.5 ± 0.5) showed better visualization on the cine images. Wilcoxon sum rank test proved the differences in the visualization of the papillary muscles and the blood pool contrast statistically significant (papillary muscles: *P* < 0.01; blood pool contrast: *P* = 0.02). RT-MRI was superior in image quality, especially in young patients with breath holding problems (Fig. 3 and supporting video in Online Supplementary Material 1, Part 2).

**Table 3:** Structure visualization rating results

Patient	Endocardial border		Epicardial border		Papillary muscles		Blood pool contrast		Myocardium		Cardiac motion		Mean ± SD	
	Cine	RT	Cine	RT	Cine	RT	Cine	RT	Cine	RT	Cine	RT	Cine	RT
1	4.0 ± 0.0	3.0 ± 0.0	3.7 ± 0.6	3.0 ± 0.0	4.0 ± 0.0	3.0 ± 0.0	4.0 ± 0.0	3.0 ± 0.0	3.3 ± 0.6	3.0 ± 0.0	4.0 ± 0.0	4.0 ± 0.0	3.8 ± 0.3	3.2 ± 0.4
2	4.0 ± 0.0	3.7 ± 0.6	4.0 ± 0.0	3.7 ± 0.6	4.0 ± 0.0	3.7 ± 0.6	4.0 ± 0.0	3.7 ± 0.6	4.0 ± 0.0	4.0 ± 0.0	4.0 ± 0.0	4.0 ± 0.0	4.0 ± 0.0	3.8 ± 0.2
3	3.3 ± 0.6	3.0 ± 0.0	3.0 ± 0.0	3.0 ± 0.0	4.0 ± 0.0	3.0 ± 0.0	3.7 ± 0.6	3.0 ± 0.0	3.7 ± 0.6	3.3 ± 0.6	4.0 ± 0.0	4.0 ± 0.0	3.6 ± 0.4	3.2 ± 0.4
4	3.0 ± 0.0	4.0 ± 0.0	3.3 ± 0.6	4.0 ± 0.0	4.0 ± 0.0	4.0 ± 0.0	4.0 ± 0.0	4.0 ± 0.0	3.7 ± 0.6	4.0 ± 0.0	4.0 ± 0.0	4.0 ± 0.0	3.7 ± 0.4	4.0 ± 0.0
5	4.0 ± 0.0	3.3 ± 0.6	4.0 ± 0.0	3.3 ± 0.6	4.0 ± 0.0	3.3 ± 0.6	4.0 ± 0.0	3.3 ± 0.6	4.0 ± 0.0	4.0 ± 4.0	4.0 ± 0.0	4.0 ± 0.0	4.0 ± 0.0	3.6 ± 0.3
6	4.0 ± 0.0	3.0 ± 0.0	4.0 ± 0.0	3.0 ± 0.0	4.0 ± 0.0	3.0 ± 0.0	4.0 ± 0.0	3.0 ± 0.0	4.0 ± 0.0	3.7 ± 0.6	4.0 ± 0.0	4.0 ± 0.0	4.0 ± 0.0	3.3 ± 0.4
7	4.0 ± 0.0	3.0 ± 0.0	4.0 ± 0.0	3.0 ± 0.0	4.0 ± 0.0	3.0 ± 0.0	4.0 ± 0.0	3.0 ± 0.0	3.3 ± 0.6	3.0 ± 0.0	4.0 ± 0.0	3.0 ± 3.0	3.9 ± 0.3	3.0 ± 0.0
8	3.3 ± 0.6	4.0 ± 0.0	4.0 ± 0.0	4.0 ± 0.0	4.0 ± 0.0	4.0 ± 0.0	4.0 ± 0.0	4.0 ± 0.0	4.0 ± 0.0	4.0 ± 0.0	4.0 ± 0.0	4.0 ± 0.0	3.9 ± 0.3	4.0 ± 0.0
9	4.0 ± 0.0	3.0 ± 0.0	4.0 ± 0.0	3.0 ± 0.0	4.0 ± 0.0	3.0 ± 0.0	4.0 ± 0.0	3.0 ± 0.0	4.0 ± 0.0	3.0 ± 0.0	4.0 ± 0.0	4.0 ± 0.0	4.0 ± 0.0	3.2 ± 0.4
10	4.0 ± 0.0	3.0 ± 0.0	3.7 ± 0.6	3.0 ± 0.0	4.0 ± 0.0	3.0 ± 0.0	4.0 ± 0.0	3.0 ± 0.0	4.0 ± 0.0	3.0 ± 0.0	4.0 ± 0.0	4.0 ± 0.0	3.9 ± 0.1	3.2 ± 0.4
11	4.0 ± 0.0	3.0 ± 0.0	4.0 ± 0.0	3.0 ± 0.0	4.0 ± 0.0	3.0 ± 0.0	4.0 ± 0.0	3.0 ± 0.0	4.0 ± 0.0	3.0 ± 0.0	4.0 ± 0.0	4.0 ± 0.0	4.0 ± 0.0	3.2 ± 0.4
12	4.0 ± 0.0	3.0 ± 0.0	4.0 ± 0.0	3.0 ± 0.0	4.0 ± 0.0	3.0 ± 0.0	4.0 ± 0.0	3.0 ± 0.0	4.0 ± 0.0	3.7 ± 0.6	4.0 ± 0.0	4.0 ± 0.0	4.0 ± 0.0	3.3 ± 0.4
13	3.0 ± 0.0	4.0 ± 0.0	3.0 ± 0.0	4.0 ± 0.0	3.3 ± 0.6	4.0 ± 0.0	3.0 ± 0.0	4.0 ± 0.0	3.0 ± 0.0	4.0 ± 4.0	3.0 ± 0.0	4.0 ± 0.0	3.1 ± 0.1	4.0 ± 0.0
14	4.0 ± 0.0	4.0 ± 0.0	4.0 ± 0.0	4.0 ± 0.0	3.7 ± 0.6	4.0 ± 0.0	4.0 ± 0.0	4.0 ± 0.0	4.0 ± 0.0	4.0 ± 4.0	4.0 ± 0.0	4.0 ± 0.0	3.9 ± 0.1	4.0 ± 0.0
15	4.0 ± 0.0	3.0 ± 0.0	4.0 ± 0.0	3.0 ± 0.0	4.0 ± 0.0	3.0 ± 0.0	4.0 ± 0.0	3.0 ± 0.0	4.0 ± 0.0	3.0 ± 0.0	4.0 ± 0.0	3.7 ± 0.6	4.0 ± 0.0	3.1 ± 0.3
16	4.0 ± 0.0	4.0 ± 0.0	4.0 ± 0.0	4.0 ± 4.0	4.0 ± 0.0	4.0 ± 0.0	4.0 ± 0.0	4.0 ± 0.0	4.0 ± 0.0	4.0 ± 4.0	4.0 ± 0.0	4.0 ± 0.0	4.0 ± 4.0	4.0 ± 0.0
17	3.0 ± 0.0	4.0 ± 0.0	3.0 ± 0.0	4.0 ± 4.0	4.0 ± 0.0	4.0 ± 0.0	4.0 ± 0.0	4.0 ± 0.0	4.0 ± 0.0	4.0 ± 4.0	4.0 ± 0.0	4.0 ± 0.0	3.7 ± 0.5	4.0 ± 0.0
18	4.0 ± 0.0	4.0 ± 0.0	4.0 ± 0.0	4.0 ± 4.0	4.0 ± 0.0	4.0 ± 0.0	4.0 ± 0.0	4.0 ± 0.0	4.0 ± 0.0	4.0 ± 4.0	4.0 ± 0.0	4.0 ± 0.0	4.0 ± 0.0	4.0 ± 0.0
19	3.0 ± 0.0	4.0 ± 0.0	3.3 ± 0.6	4.0 ± 0.0	3.7 ± 0.6	4.0 ± 0.0	3.0 ± 0.0	4.0 ± 0.0	3.0 ± 0.0	4.0 ± 4.0	3.7 ± 0.6	4.0 ± 0.0	3.3 ± 0.3	4.0 ± 0.0
20	3.0 ± 0.0	3.0 ± 0.0	3.3 ± 0.6	3.0 ± 0.0	4.0 ± 0.0	3.0 ± 0.0	4.0 ± 0.0	3.0 ± 0.0	4.0 ± 0.0	3.0 ± 3.0	4.0 ± 0.0	3.7 ± 0.6	3.7 ± 0.4	3.1 ± 0.3
Mean ± SD	3.7 ± 0.4	3.5 ± 0.5	3.7 ± 0.4	3.5 ± 0.5	3.9 ± 0.2	3.5 ± 0.5	3.9 ± 0.3	3.5 ± 0.5	3.8 ± 0.3	3.6 ± 0.5	3.9 ± 0.2	3.9 ± 0.2	3.8 ± 0.7	3.6 ± 0.4
Wilcoxon *P*	0.221		0.117		0.002		0.022		0.107		0.783		0.050	

Results are demonstrated for the comparison of standard cine MRI and RT-MRI

Image quality rating scale: 1 = no visibility, 2 = poor, 3 = good, 4 = excellent

*MRI *magnetic resonance imaging*, RT* real-time, *SD* standard deviation

#### Artifact rating (European cardiac MR registry)

The two most prevalent artifacts were metallic artifacts and respiratory ghosting, whereas wrap-around, cardiac ghosting, blurring/miss-triggering, and shimming artifacts were rare on standard cine and RT images (Fig. 2).

While cine images, especially in younger patients often showed respiratory artifacts on multiple slices (1.5 ± 1.4), respiratory artifacts did not occur in RT-MRI (0.0 ± 0.0). Wilcoxon rank sum test proved the increased incidence of respiratory ghosting on cine MRI statistically significant (*P* < 0.01).

Metallic artifacts from sternal wires, Contegra conduits and coils from occlusion of patent ductus arteriosus occurred on RT images (1.2 ± 1.3) and cine images (1.8 ± 1.4) but showed a significantly higher artifact extent on the cine images (*P* < 0.01) (Fig. 2 and supporting video in Online Supplementary Material 2).

The overall evaluation of the artifact rating demonstrated that RT images were significantly less affected by artifacts than cine images (cine: 0.5 ± 0.4; RT: 0.2 ± 0.2; *P* < 0.01).

### Ventricular volumetry

#### Left ventricle

**Fig. 4:**
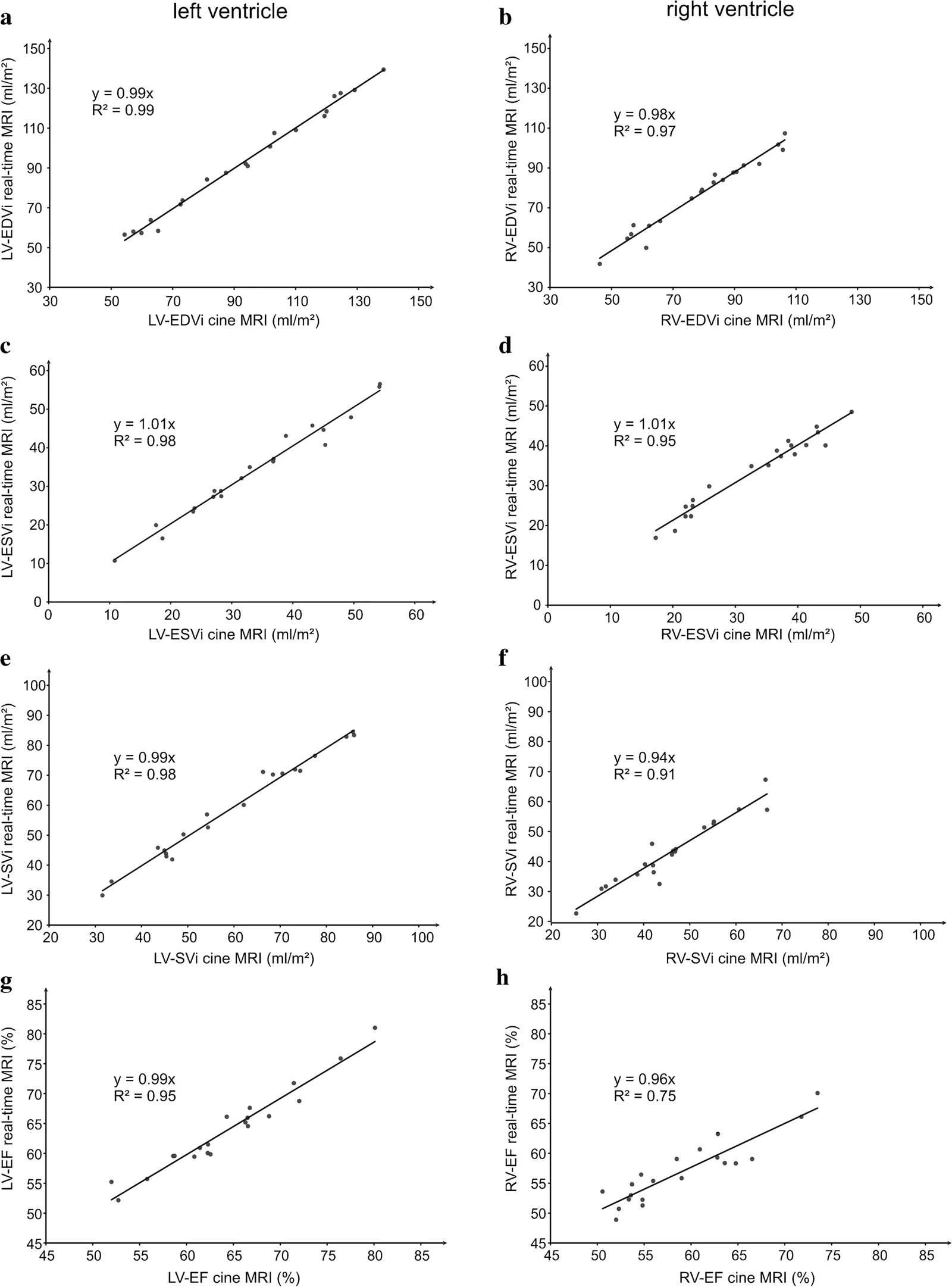
Linear regression analysis. Linear regression analyses for LV (**a, c, e, g**) and RV (**b, d, f, h**) volumetric measurements in cine MRI and RT-MRI revealed good correlations in EDVi (ml/m^2^), ESVi (ml/m^2^), SVi (ml/m^2^) and EF (%). The regression lines, corresponding linear equations and coefficients of determination are integrated into the graphs. *EDVi* end-diastolic volume indexed to body surface area*, EF* ejection fraction, *ESVi* end-systolic volume indexed to body surface area*, LV* left ventricle*, RT-MRI* real-time magnetic resonance imaging*, RV* right ventricle, *MRI* magnetic resonance imaging, *SVi* stroke volume indexed to body surface area

Linear regression analyses yielded good correlations between the two methods for the left ventricular end-diastolic volume indexed to body surface area (LV-EDVi), the left ventricular end-systolic volume indexed to body surface area (LV-ESVi), the left ventricular stroke volume indexed to body surface area (LV-SVi) and the left ventricular ejection fraction (LV-EF) (Fig. 4).

**Fig. 5:**
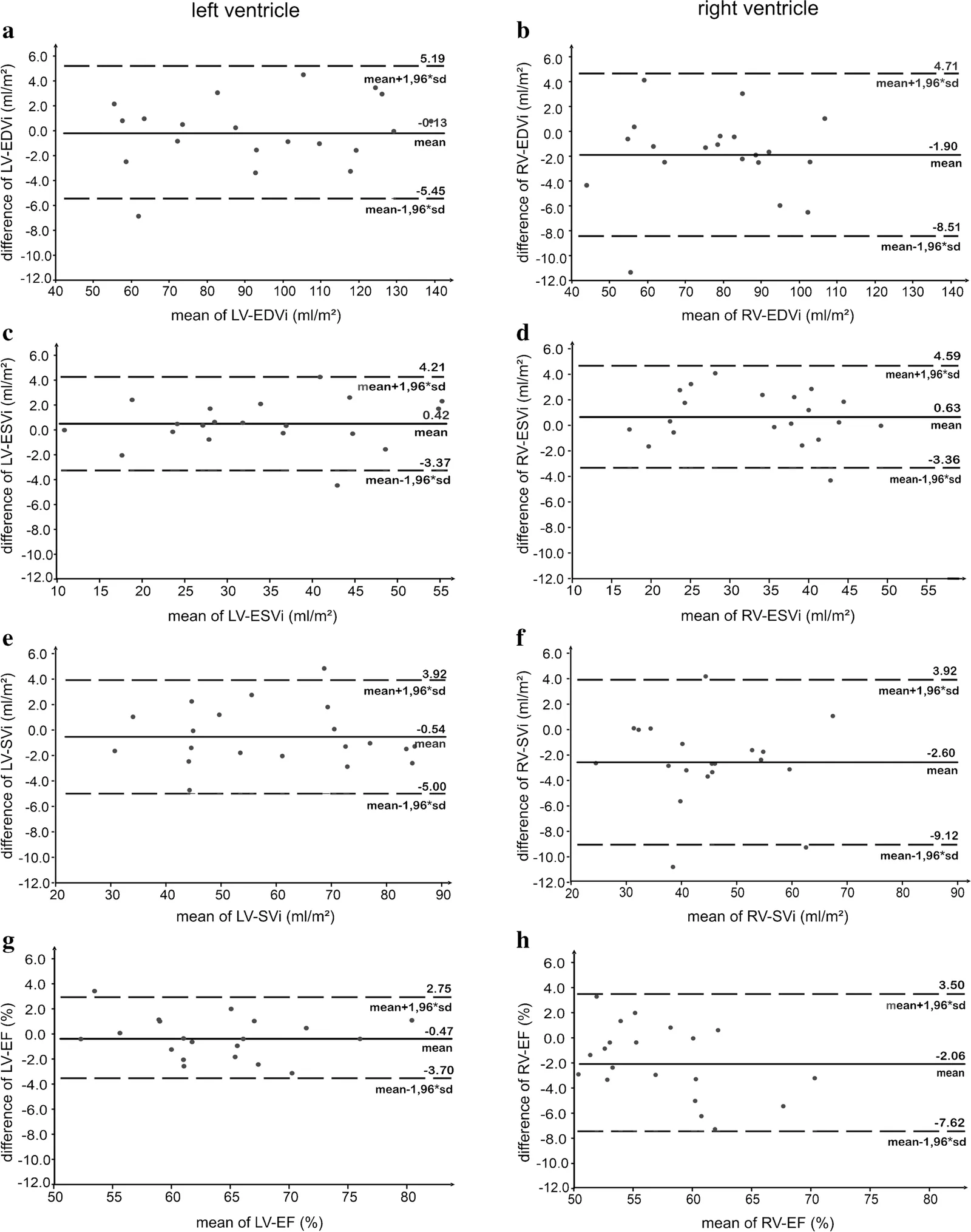
Bland–Altman plots. Bland–Altman plots for LV (**a, c, e, g**) and RV (**b, d, f, h**) demonstrate the calculated difference (RT-MRI – cine MRI) on the y-axis as a function of the mean value from both imaging techniques on the x-axis. Solid lines represent the mean value of the differences (RT-MRI – cine MRI); dashed lines show the mean difference ± 1.96 standard deviation. *LV* left ventricle*, MRI* magnetic resonance imaging, *RT-MRI* real-time magnetic resonance imaging, *RV* right ventricle

Bland–Altman analysis showed good agreements between RT-MRI and cine MRI for the results of LV-EDVi (LoA = -0.1 ml/m^2^ ± 2.7 ml/m^2^), LV-ESVi (LoA = 0.4 ml/m^2^ ± 1.9 ml/m^2^), LV-SVi (LoA = -0.5 ml/m^2^ ± 2.3 ml/m^2^) and LV-EF (LoA = -0.5% ± 1.6%) (Fig. 5).

A paired sample *t*-test proved no statistically significant differences between standard cine MRI and RT-MRI were found for the LV-EDVi (*P* = 0.83), LV-ESVi (*P* = 0.34), LV-SVi (*P* = 0.30) and LV-EF (*P* = 0.21) (Table 4).

**Table 4:** Mean ventricular volumetry results

	Cardiac function parameters	Cine MRI	RT-MRI	Paired samples t-test (*P*-value)	Intraclass correlation coefficient	
					Inter-rater reliability (cine / RT)	Intra-rater reliability (cine / RT)
Left ventricle	EDVi (ml/m^2^)	93.6 ± 26.8	93.4 ± 27.4	0.83	1.00 / 0.99	1.00 / 1.00
	ESVi (ml/m^2^)	33.7 ± 12.3	34.1 ± 12.5	0.34	0.99 / 0.99	1.00 / 0.99
	SVi (ml/m^2^)	59.9 ± 17.2	59.3 ± 17.1	0.30	0.99 / 0.99	0.99 / 0.99
	EF (%)	64.3 ± 7.3	63.9 ± 7.0	0.21	0.99 / 0.99	0.99 / 0.99
Right ventricle	EDVi (ml/m^2^)	79.0 ± 18.4	77.1 ± 18.4	0.02	0.99 / 0.99	0.99 / 0.99
	ESVi (ml/m^2^)	32.8 ± 9.7	33.4 ± 9.4	0.18	0.99 / 0.99	0.99 / 0.99
	SVi (ml/m^2^)	46.2 ± 11.3	43.6 ± 11.0	0.00	0.99 / 0.99	0.99 / 0.99
	EF (%)	59.0 ± 6.6	56.9 ± 5.3	0.00	0.96 / 0.86	0.98 / 0.98

Results are demonstrated for the left and right ventricular function parameters obtained with standard cine MRI and RT-MRI

*EDVi* end-diastolic volume indexed to body surface area, *EF* ejection fraction, *ESVi* end-systolic volume indexed to body surface area, *RT* real-time, *RT-MRI* real-time magnetic resonance imaging, *SVi* stroke volume indexed to body surface area

#### Right ventricle

Linear regression analyses showed good correlations between RT-MRI and cine MRI for the right ventricular end-diastolic volume indexed to body surface area (RV-EDVi), the right ventricular end-systolic volume indexed to body surface area (RV-ESVi), the right ventricular stroke volume indexed to body surface area (RV-SVi) and the right ventricular ejection fraction (RV-EF) (Fig. 4).

The Bland–Altman analysis demonstrated good agreements between RT-MRI and cine MRI for the results of RV-EDVi (LoA = -1.9 ml/m^2^ ± 3.4 ml/m^2^), RV-ESVi (LoA = 0.6 ml/m^2^ ± 2.0 ml/m^2^), RV-SVi (LoA = -2.6 ml/m^2^ ± 3.3 ml/m^2^) and RV-EF (LoA = -2.1% ± 2.8%) (Fig. 5).

Nevertheless, the differences for the RV-EDVi, RV-ESVi, RV-SVi and RV-EF between cine MRI and RT-MRI were greater compared to the LV volume results (Table 4) with lower coefficients of determination from linear regression analyses and lower LoA in Bland–Altman plots (Figs. 4 and 5).

Paired sample *t*-tests proved the differences between both methods statistically significant with lower values on average for RT-MRI in RV-EDVi (*P* = 0.02), RV SVi (*P* < 0.01) and RV-EF (*P* < 0.01). No significant differences were found for the RV-ESVi (*P* = 0.18) (Table 4).

#### Reproducibility

The results for the ICC were good to very good (ICC = 0.86–1.00) for the left and right ventricular EDVi, ESVi, SVi and EF evaluation from the cine MRI and RT-MRI for inter- and intra-rater reliability (Table 4).

## Discussion

This study demonstrates the feasibility of studying cardiac volumetry with RT-MRI during free breathing in pediatric patients. Implementing user-friendly, time-saving software for RT-MRI postprocessing enabled us to integrate this method in everyday clinical practice. Retrospective respiratory binning guaranteed image stabilization and high image quality. Thus, quantitative volume evaluation was enabled from data acquired under physiological conditions from almost all patients in the study. Cardiac function could not be analyzed in one patient because of an unusable respiratory bellows signal.

Although the benefits of RT-MRI are well known, the lack of quantitative evaluation strategies that offer fast post-processing comparable to standard cine MRI precludes its current use in clinical practice. We were able to reduce the time for post-processing by implementing a user-friendly GUI. Thus, the additional net time required was reduced to 2 min.

At present, cine MRI with slice per slice data acquisition and repetitive breath holding is the clinical standard for the examination of cardiac volumetry. In cases where breath holding is impossible due to preexisting conditions or patient age, cine MR images may be acquired during free breathing, which is associated with significantly poorer image quality. For patients with heart or lung diseases, and especially for children, breath holding is a major disadvantage that reduces the patient’s comfort [5], induces additional anxiety [22] and is unreliable for small children and infants. RT-MRI enables image acquisition during free breathing with simultaneous high image quality. The fact that the youngest participant in the study fell asleep during RT-MRI emphasizes the great advantages of free breathing. This indicates that cardiac volumetry based on RT-MRI is likely suitable for sleep and feeding methods in neonates and infants as established for other applications such as brain MRI [23] or for cardiac MRI and flow measurements that do not require breath holding [24, 25]. Potentially eliminating sedation or general anesthesia could reduce the resources needed for imaging and would offer major patient safety benefits, as both pose short- and long-term risks [26]. In addition, general anesthesia and sedation impair cardiac function. Anesthesia with mechanical ventilation based on positive intrathoracic pressure completely reverses the physiological respiratory influence on cardiac function [27].

In our study, problems with breath holding often resulted in respiratory ghosting on cine images, reducing image quality. This has been demonstrated in previous studies [13]. In our study cine MRI was slightly superior in structure visualization in adolescent patients, RT-MRI acquired during free breathing achieved better ratings for the structure visualization in younger children with breath-holding problems.

RT-MRI provided a significantly better robustness against metallic artifacts. This can be explained by a more rapid data acquisition with radial (i.e. frequency-encoding) trajectories in comparison to cine MRI with a Cartesian phase-encoding scheme [6–8]. Metallic implants interfere with MRI by changing the phase of the MRI signal. B-SSFP sequences are very sensitive to phase errors as they superimpose differently affected gradient and RF-refocused echoes. However, image artifacts reduce if the total image acquisition time decreases to only 33 ms as in the present study and the use of spatial phase-encoding is avoided. Thereby, RT-MRI appears to be the preferred imaging technique for children with metallic stents or sternal clips.

Compressed sensing techniques are commonly used for accelerated imaging techniques and already used in RT cine cardiovascular MRI, thereby improving temporal resolution while reducing image quality [28]. Mathematically, compressed sensing only solves a linear inverse problem, that is insufficient for image reconstruction of undersampled cardiac MRI. In contrast, the RT-MRI used in this study does not use compressed sensing. Here, the computation is performed using a nonlinear inverse reconstruction. This allows the complex MRI image and all coil profiles to be computed simultaneously. In this way, the images of the heart can be obtained in RT while maintaining high image quality.

LV and RV volumes showed good correlations between the RT images acquired during free breathing and binned corresponding to a low lung volume and cine images with a slice per slice acquisition in end-expiratory breath holding. No significant differences were found between either technique for the LV-EDVi, LV-ESVi, LV-SVi and LV-EF. This is in line with other comparative MR studies [13, 29]. However, the values for RV-EDVi, RV-SVi and RV-EF calculated from the RT-MRI acquired during free breathing and binned according to a low lung volume were significantly lower.

Previous RT-MRI studies during free-breathing demonstrated that even with calm breathing, respiration influences cardiac function [9, 10, 30]. The RV was especially affected significantly [9, 10].

The influence of breathing on RV function can be explained by heart–lung interactions [31, 32]. The right ventricular preload is influenced significantly by the respiratory phase. In the end-expiratory phase during free breathing, less negative intrathoracic pressure results in less venous return to the right heart [31, 32]. As explained by the Frank Starling law, the reduced RV preload results in a lower RV stroke volume [33, 34].

In our study, we observed lower values for RV-EDVi, RV-SVi and RV-EF derived from RT volumetry, acquired during free-breathing and binned corresponding to a low lung volume in comparison to results from cine MRI during end-expiratory breath holding. Differences in RV function between the same respiratory phase during breath holding and free-breathing have been described in a previous RT-MRI study [10], highlighting the substantial physiological influence of cardiopulmonary interaction on RV function and emphasizing that breath holding is an unphysiological condition.

### Limitations and plans for future

Even though our study demonstrates the feasibility of cardiac RT-MRI volumetry during free breathing in pediatric patients, some limitations must be considered:

First, our study included pediatric patients ages 5 to 17 years. Future studies will have to demonstrate the feasibility of RT-MRI during free breathing to reduce the need for sedation or even anesthesia in preschool children and to enable cardiac volumetry testing in neonates and infants in combination with sleep and feed studies.

Second, respiratory bellows do not provide quantitative data for lung volume and breathing depths. Thus, the heart position varied in images with equal respiratory bellows values resulting in greater movement from image to image in RT-MRI. MR-compatible spirometry could provide quantitative information on respiratory flow in future studies [9].

Third, the poor quality of the respiratory bellows made it necessary to define the end-expiratory breathing class very broadly and the class was solely defined based on absolute respiratory bellows values without consideration of respiratory flow. This resulted in less image stabilization and the respiratory influence on cardiac volumes was probably underestimated.

Fourth, volumes were obtained from 2-D short axis stacks. In the future, volume determinations of 3-D data sets might provide results that are even more precise. Previous studies have already demonstrated the feasibility of real-time 3-D flow MRI [35].

Fifth, temporal resolution of RT-MRI with b-SSFP contrast at 1.5 T is limited to 33 ms. In small children with faster heart rates, the limited temporal resolution could be insufficient. Using spoiled fast low-angle shot (FLASH) contrast at 3 T would provide a temporal resolution of even 20 ms, i.e. 50 frames per s, that could provide sufficient temporal resolution for faster heart rates in infants.

Sixth, minor differences in pixel size may have resulted in minor differences between RT and conventional MR images.

## Conclusion

In comparison to cardiac cine MRI with breath holding, the combination of free-breathing RT-MRI with retrospective respiratory binning using a custom user-friendly software program provides good image quality, fewer image artifacts and, most importantly, offers a reliable clinical tool for the accurate quantification of ventricular volumes during preserved physiological conditions in pediatric patients.

## Supplementary Information

Below is the link to the electronic supplementary material.

Supplementary file1 (MP4 15543 KB) *Supporting Video: Image Quality: *The first part of the video shows a heart cycle in a midventricular slice from standard cine MRI during end-expiratory breath holding and the corresponding free-breathing RT-MRI with retrospective respiratory binning in a 15-year-old patient (patient 10) with good breath holding. The second part of the video demonstrates a heart cycle in a midventricular slice from standard cine MRI in a 5-year-old patient (patient 19.) with breath-holding problems and the corresponding RT-MRI acquired during free breathing and binned retrospectively corresponding to a low lung volume.

Supplementary file2 (MP4 9489 KB) *Supporting Video: Metallic Artifacts: *The video shows metallic artifacts from sternal wires on a midventricular slice from standard cine MRI during end-expiratory breath holding and on the corresponding midventricular slice from RT-MRI acquired during free breathing and binned retrospectively corresponding to a low lung volume. The higher artifact extent on cine MRI is obvious.
